# Supplementary figures and images for: Phenotype discovery from population brain imaging
Source: Med Image Anal. 2021 Jul;71:102050. doi: 10.1016/j.media.2021.102050 (PMC8850869; doi:10.1016/j.media.2021.102050)

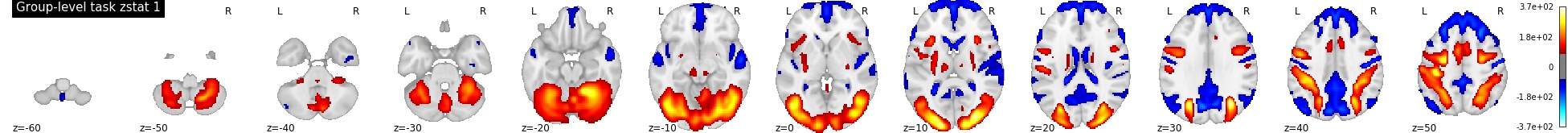

Supplement: Supplementary Data S5 — Supplementary Raw Research Data. This is open data under the CC BY license http://creativecommons.org/licenses/by/4.0/ [file mmc5.zip › mmc5.jpg]

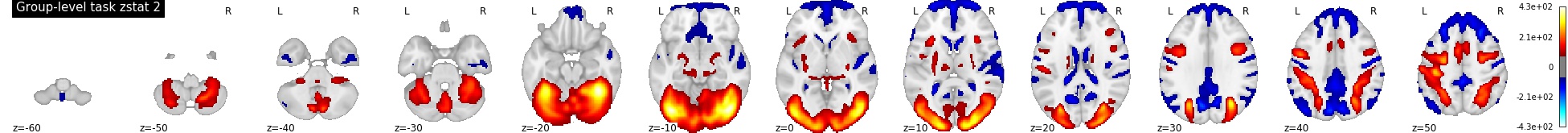

Supplement: Supplementary Data S6 — Supplementary Raw Research Data. This is open data under the CC BY license http://creativecommons.org/licenses/by/4.0/ [file mmc6.zip › mmc6.jpg]

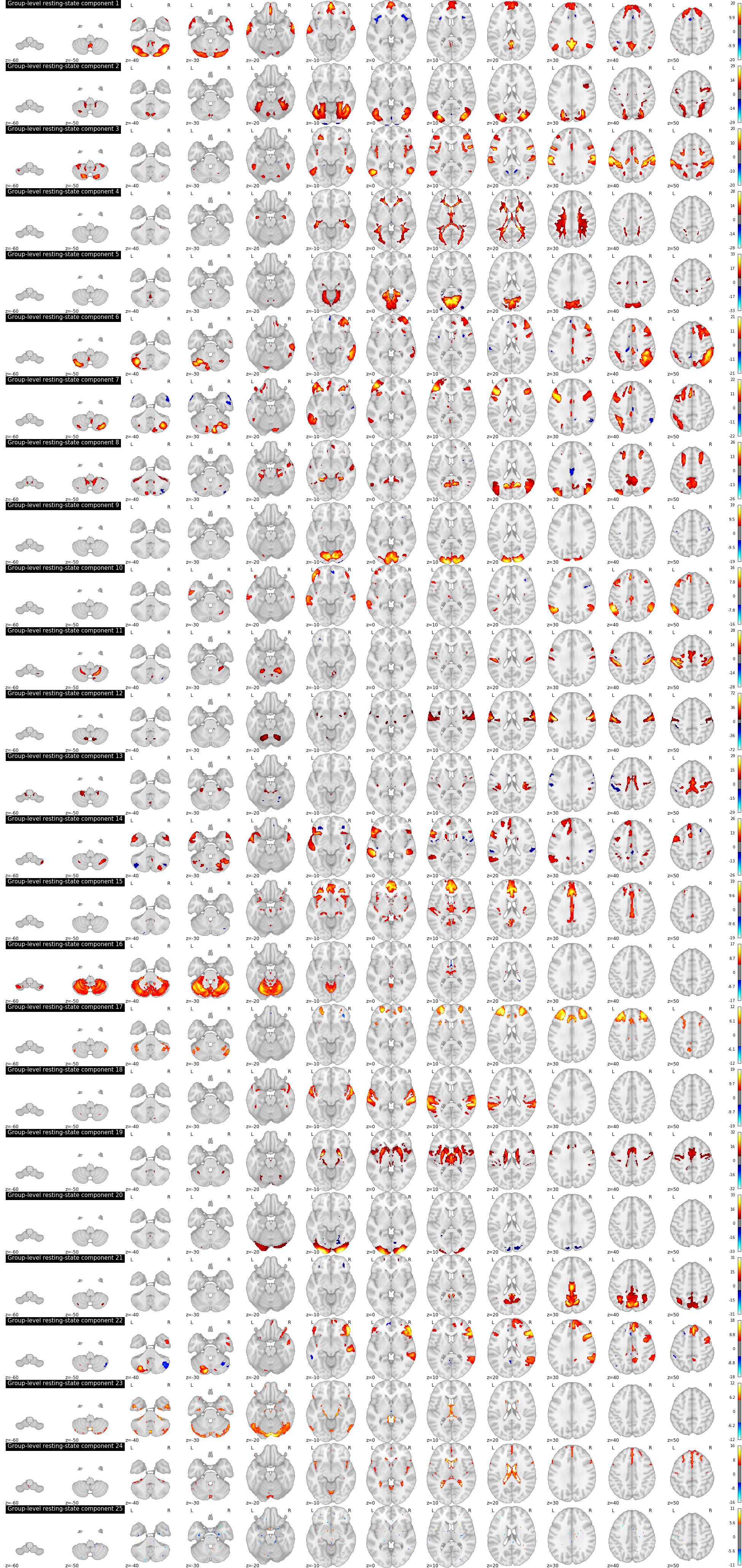

Supplement: Supplementary Data S7 — Supplementary Raw Research Data. This is open data under the CC BY license http://creativecommons.org/licenses/by/4.0/ [file mmc7.zip › mmc7.jpg]

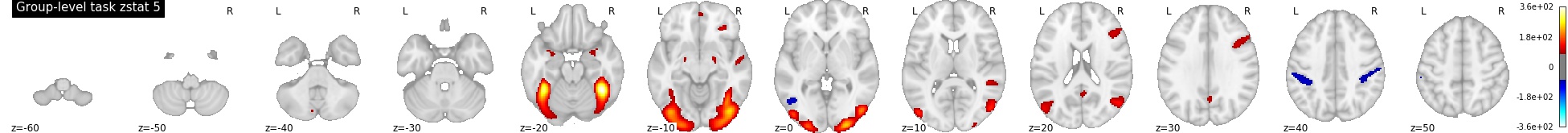

Supplement: Supplementary Data S8 — Supplementary Raw Research Data. This is open data under the CC BY license http://creativecommons.org/licenses/by/4.0/ [file mmc8.zip › mmc8.jpg]
