# Supplementary material for: Phenotype discovery from population brain imaging
Source: Med Image Anal. 2021 Jul;71:102050. doi: 10.1016/j.media.2021.102050 (PMC8850869; doi:10.1016/j.media.2021.102050)
Supplement: Supplementary Data S9 — Supplementary Raw Research Data. This is open data under the CC BY license http://creativecommons.org/licenses/by/4.0/ [file mmc9.pdf]

Appendix A. Supplementary Materials

Appendix A.1. Confounding variables regressed out in our analysis

UKB dataset: age, age squared, age X sex, age squared X sex, age (quantile normalised), age squared (quantile normalised), age X sex (quantile normalised), age squared X sex (quantile normalised), rfMRI head motion, tfMRI head motion, head size scaling, rfMRI head motion squared, tfMRI head motion squared, [4] confounds relating to bed position in scanner (x), [4] confounds relating to bed position in scanner (y), [4] confounds relating to bed position in scanner (z), [4] confounds relating to bed position in scanner (table), [4] confounds relating to bed position in scanner (x) squared, [4] confounds relating to bed position in scanner (y) squared, [4] confounds relating to bed position in scanner (z) squared, [4] confounds relating to bed position in scanner (table) squared, [10] confounds modelling slow date-related drift 1, [10] confounds modelling slow date-related drift 2, [10] confounds modelling slow date-related drift 3, [10] confounds modelling slow date-related drift 4, [10] confounds modelling slow date-related drift 5, [10] confounds modelling slow date-related drift 6, [10] confounds modelling slow date-related drift 7, [10] confounds modelling slow date-related drift 8, [10] confounds modelling slow date-related drift 9, [10] confounds modelling slow date-related drift 10, rfMRI head motion (quantile normalised), tfMRI head motion (quantile normalised), head size scaling (quantile normalised), [4] confounds relating to bed position in scanner (x) (quantile normalised), [4] confounds relating to bed position in scanner (y) (quantile normalised), [4] confounds relating to bed position in scanner (z) (quantile normalised), [4] confounds relating to bed position in scanner (table) (quantile normalised), [4] confounds relating to bed position in scanner (x) squared (quantile normalised), [4] confounds relating to bed position in scanner (y) squared (quantile normalised), [4] confounds relating to bed position in scanner (z) squared (quantile normalised), [4] confounds relating to bed position in scanner (table) squared (quantile normalised), [10] confounds modelling slow date-related drift 1 (quantile normalised), [10] confounds modelling slow date-related drift 2 (quantile normalised), [10] confounds modelling slow date-related drift 3 (quantile normalised), [10] confounds modelling slow date-related drift 4 (quantile normalised), [10] confounds modelling slow date-related drift 5 (quantile normalised), [10] confounds modelling slow date-related drift 6 (quantile normalised), [10] confounds modelling slow date-related drift 7 (quantile normalised), [10] confounds modelling slow date-related drift 8 (quantile normalised), [10] confounds modelling slow date-related drift 9 (quantile normalised), [10] confounds modelling slow date-related drift 10 (quantile normalised), imaging centre, sex.

HCP dataset: image reconstruction version, age, age squared, sex, age X sex, age squared X sex, race, ethnicity, rfMRI motion, Height, Weight, FS_IntraCranial_Vol, FS_BrainSeg_Vol.

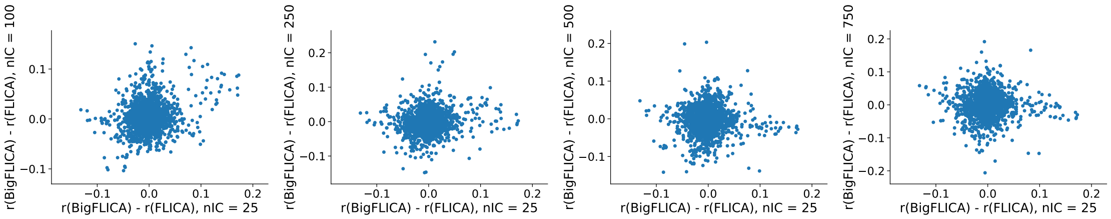

Figure A.6: The relationship between the difference of prediction accuracy of BigFLICA and FLICA across different number of components.

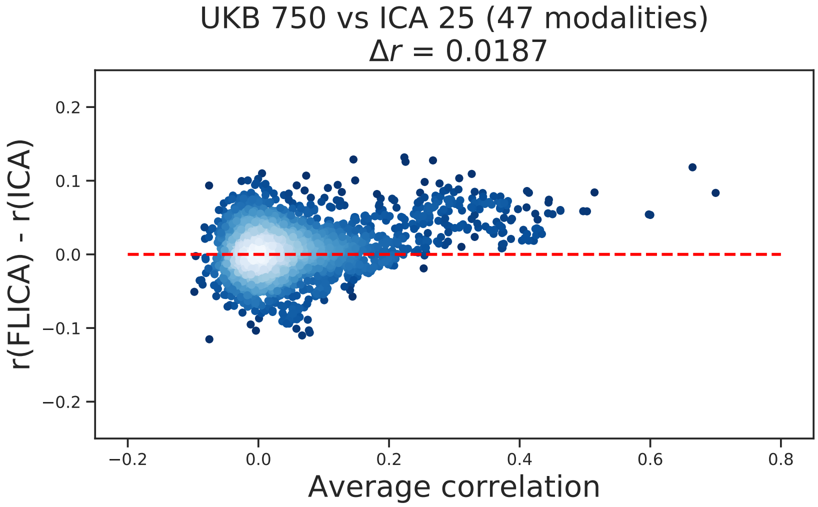

Figure A.7: Comparing the prediction performance of the 750-dimensional FLICA with the 25-dimensional single-modality ICA concatenated across 47 modalities in the UKB data.

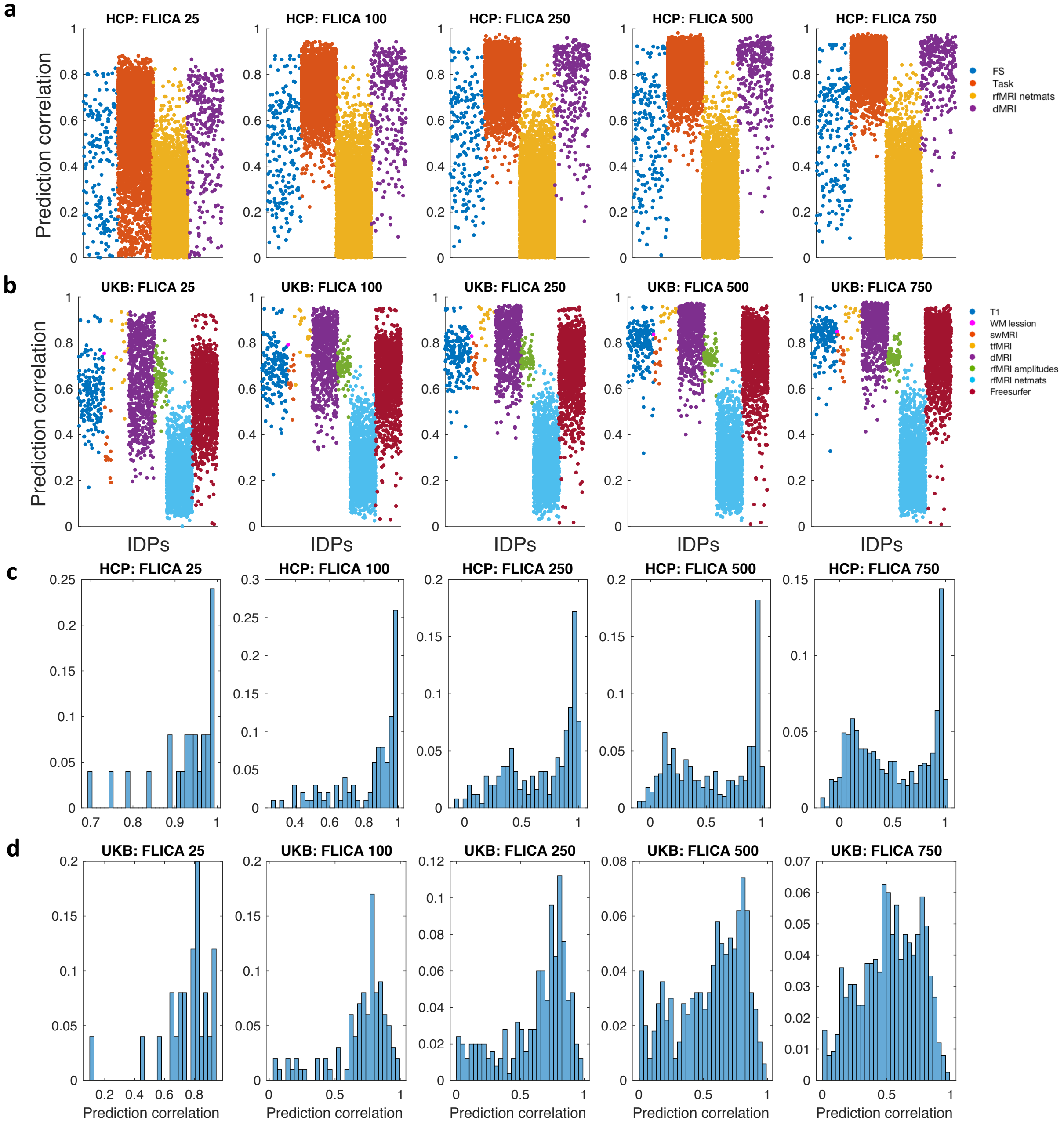

Figure A.8: Relationships between FLICA and IDPs. **(a,b)** The plots show the results of predicting each IDP using BigFLICA modes in **a** the HCP and **b** the UKB dataset. The IDPs are appearing in order along the x axis, and are grouped and coloured by modality types. **(a,b)** The histograms of predicting BigFLICA modes using all IDPs in **c** the HCP and **d** the UKB dataset.

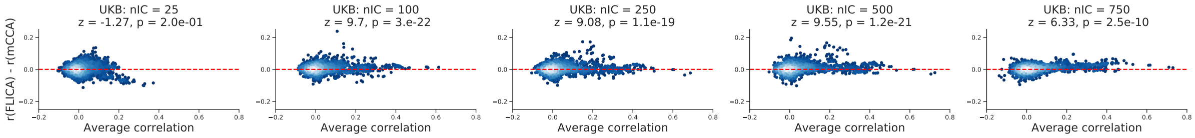

Figure A.9: Comparing FLICA and mCCA in the UKB data. Comparing the predictive performance of FLICA with mCCA (or equivalently the subject-by-component matrix obtained in the mMIGP step) across different numbers of extracted components in the UKB dataset. The FLICA and mCCA dimensions are the same in each figure.

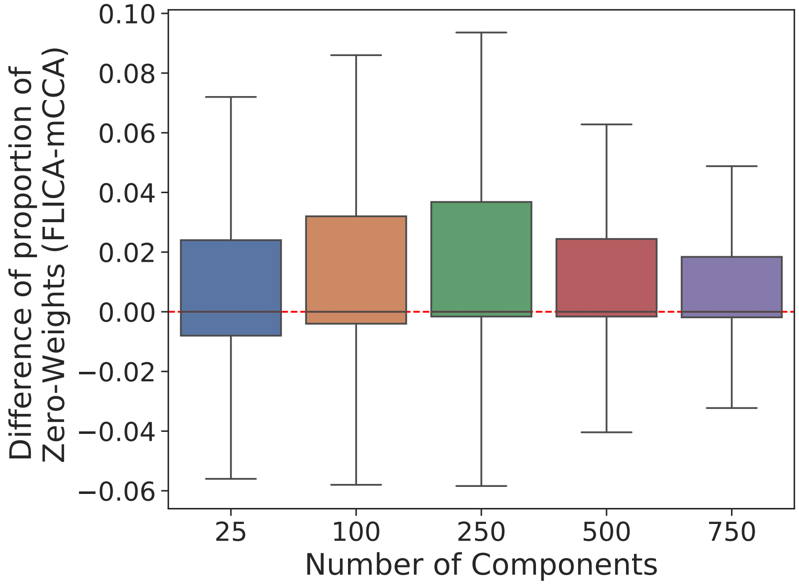

Figure A.10: The difference of the proportion of zeros weights (BigFLICA-mCCA) in predicting nIDPs across 5 dimensions of decomposition in the UKB data.

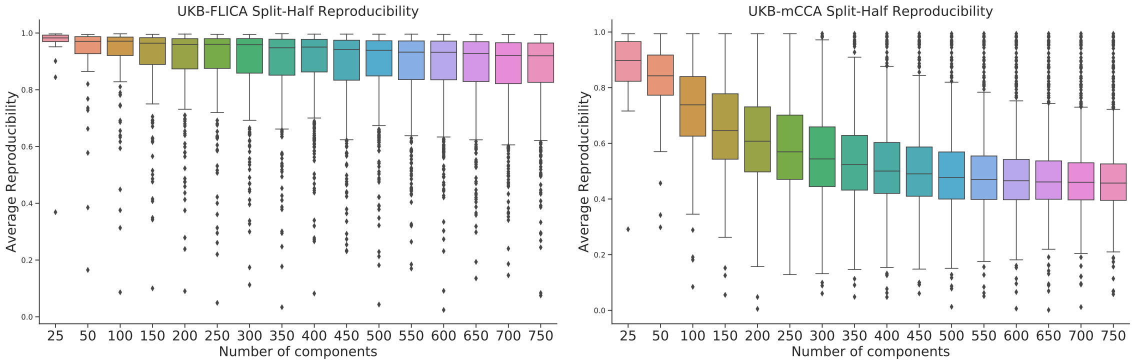

Figure A.11: Split-half reproducibility of BigFLICA and mCCA spatial maps in the UKB dataset. The split-half reproducibility of BigFLICA and mCCA in the UKB dataset by first computing the correlation between modality-wise concatenated spatial maps after eliminating low-weight voxels and then greedy matching.

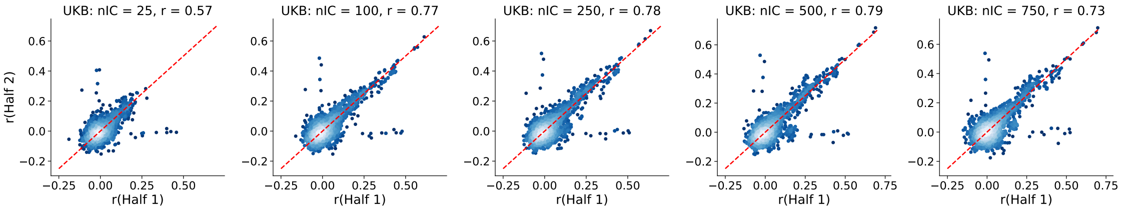

Figure A.12: Split-half prediction accuracy of all nIDPs of BigFLICA in the UKB dataset.

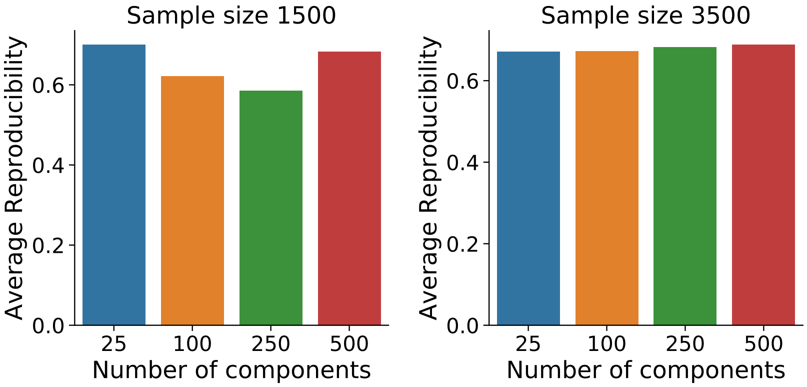

Figure A.13: Split-half reproducibility of BigFLICA using different sample size in the UKB dataset. Left: sample size = 1500. Right: sample size = 3500.

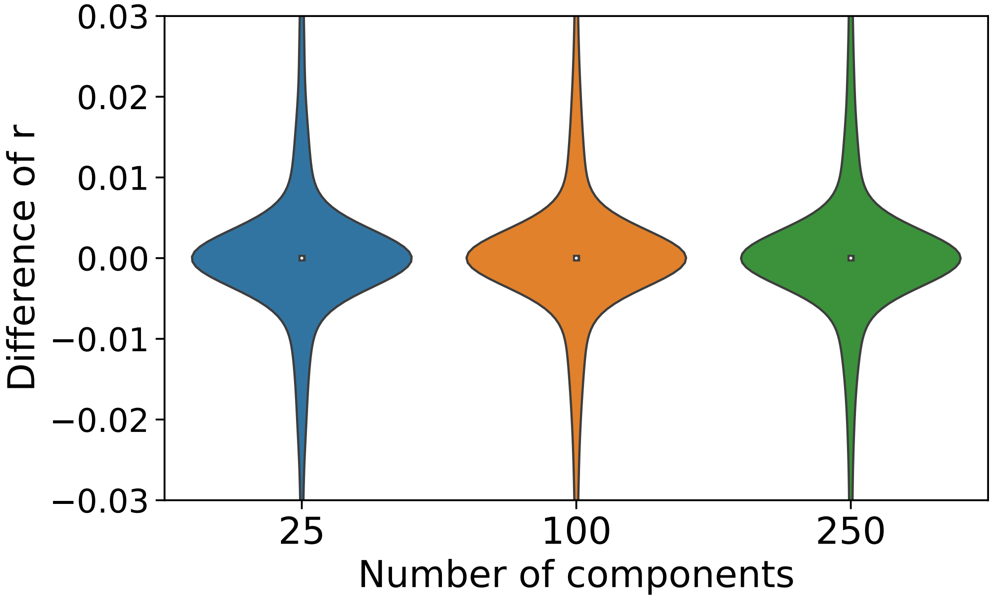

Figure A.14: Stability of prediction accuracy of BigFLICA against different train-test subject splits. We estimated the prediction correlation of each nIDP using 5 different train-test subject splits, and calculated the mean accuracy of prediction correlations for each nIDPs. We then computed the difference of the mean prediction correlation and the prediction correlation of one of the five random predictions across different number of components.

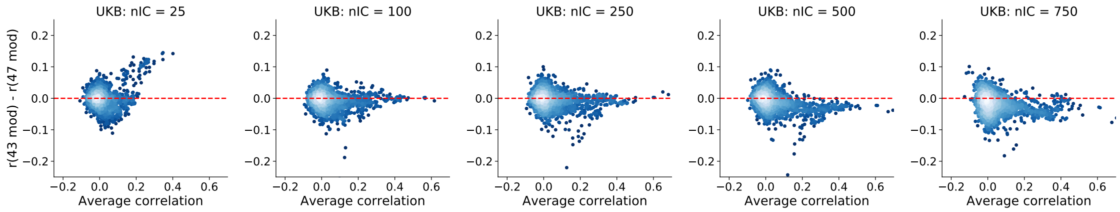

Figure A.15: Comparison of prediction accuracy of nIDPs when removing 4 noise resting-state modalities with including them in the model. An increased prediction accuracy was observed for some nIDPs in the 25-dimensional decomposition when compared with using all 47 modalities, and a similar prediction accuracy was observed for 100- and 250-dimensional decomposition, while a decreased prediction accuracy was observed for 500- and 750-dimensional decompositions.

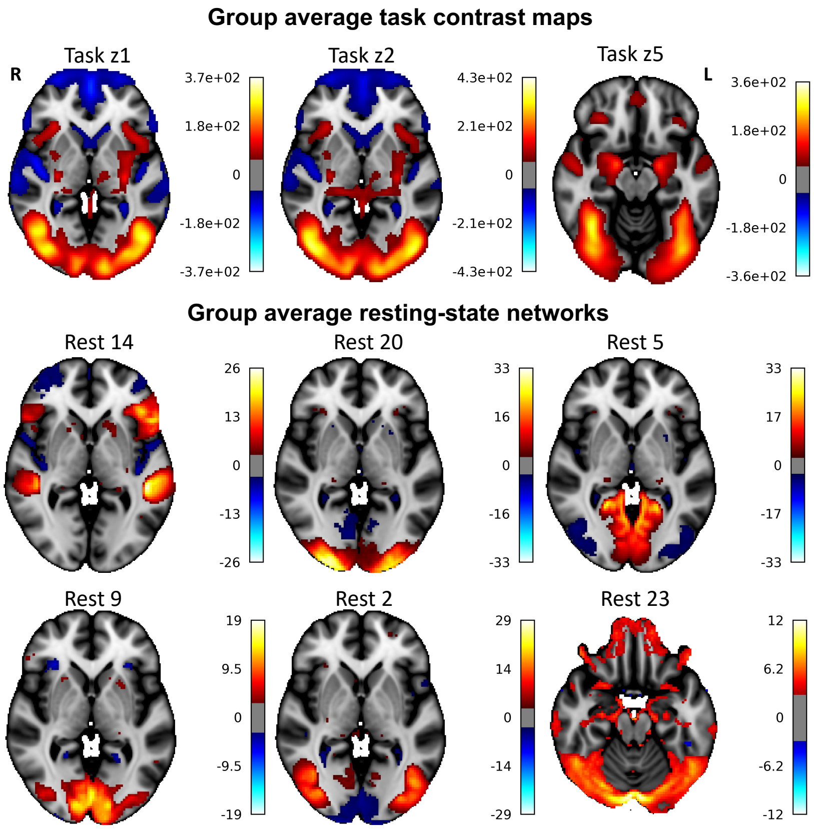

Figure A.16: Group average maps of task activations and resting-state networks in the UKB dataset. These are provided to help interpret the population variability maps (modulations of these maps) shown in **Fig. 5**. Top: group average of emotion task activation z-statistic maps (task z1: "shapes", task z2: "face", task z5: "faces>shapes"). Group average task contrast (effect size) maps c1, c2 and c5 are highly similar to z-stat maps so that they are not shown. Bottom: group average resting-state networks from a 25-dimensional ICA parcellation in the UKB data. The six maps shown here are the networks from **Fig. 5**.

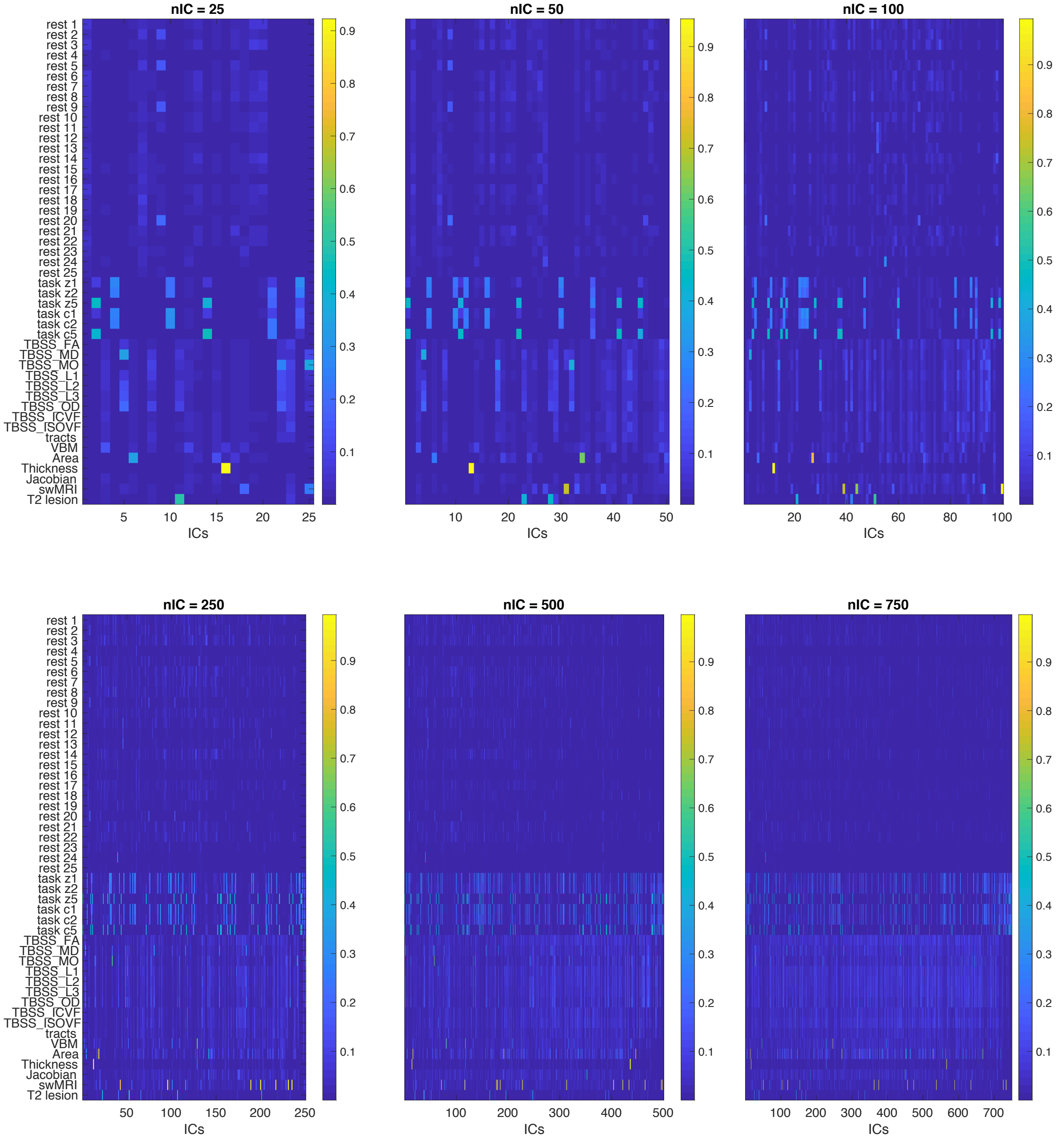

Figure A.17: The contribution of each modality in each BigFLICA mode (independent component).

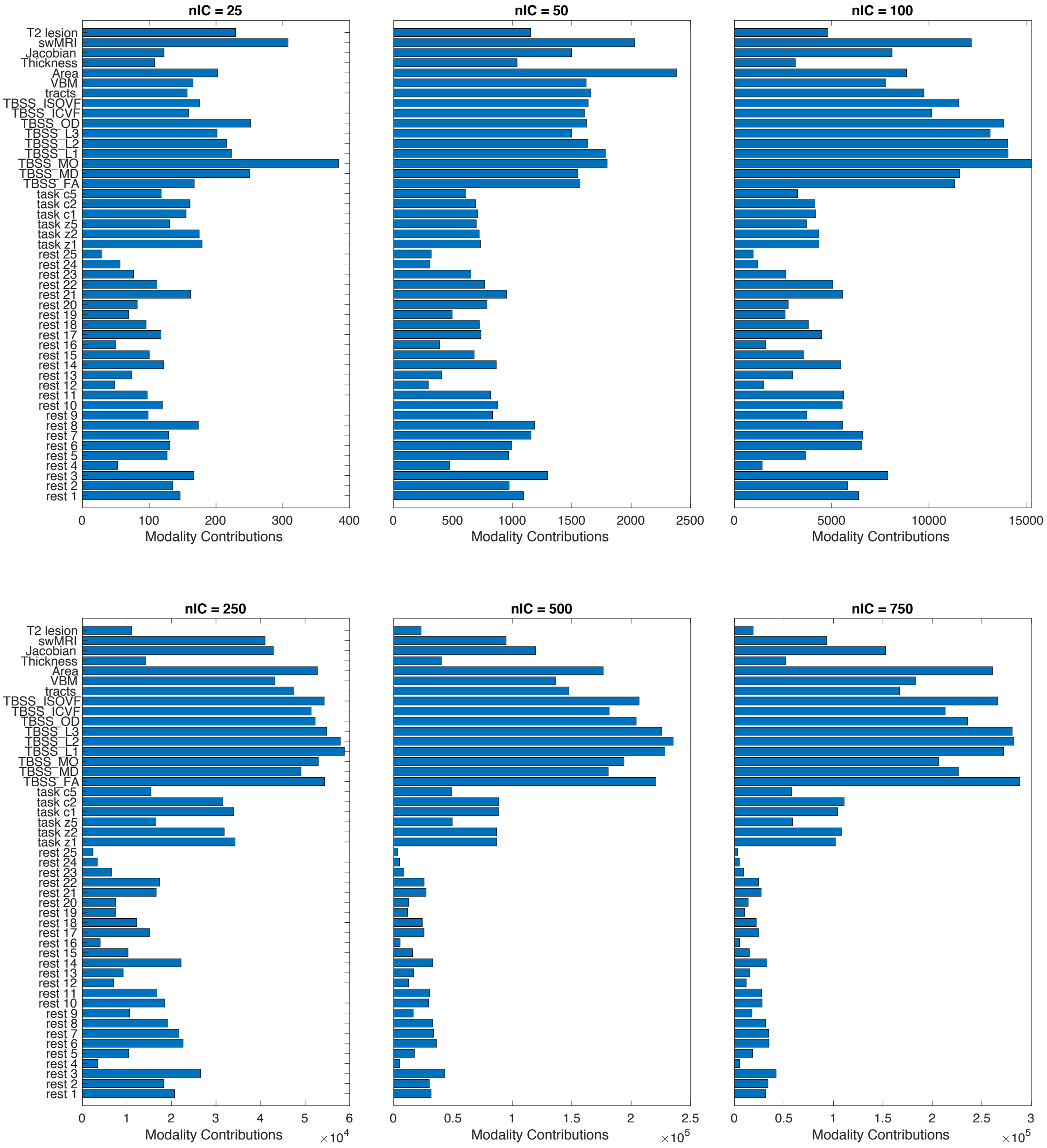

Figure A.18: The relative contribution of different modalities of a BigFLICA decomposition (nIC=25-750) in the UKB data. For each modality, we take the sum of its overall contribution (estimated by BigFLICA) across all components.

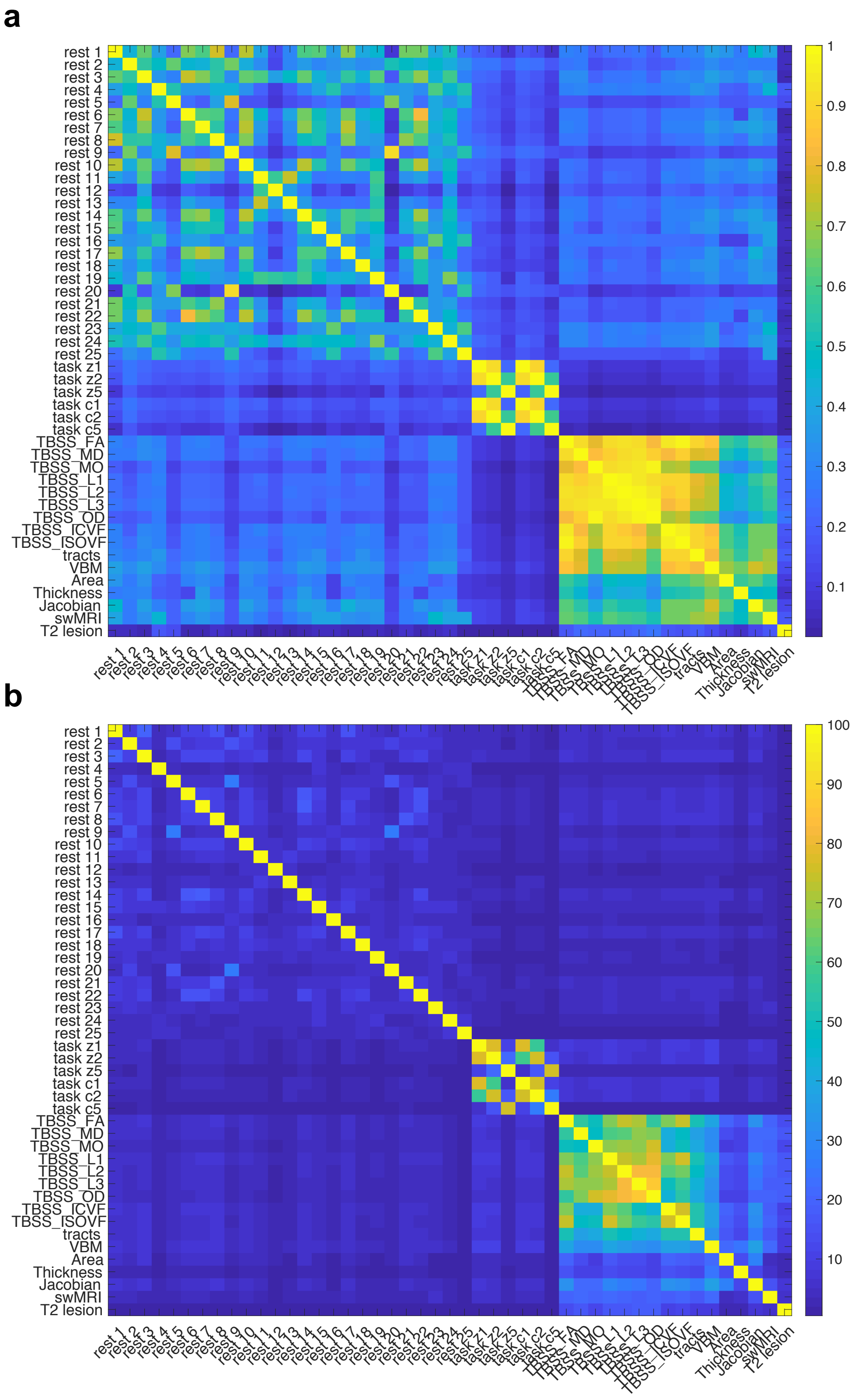

Figure A.19: The relationships between different modalities in the UKB data. a). The cosine similarity of modality contributions across 750 components (estimated by BigFLICA) between every pair of modalities. b). The amount of shared variance between two 50-dimensional single-modality ICA decompositions in each pair of modalities.

Table A.2: Comparison of prediction performance of **Cognitive Phenotypes** and **Health and Medical History Health Outcomes** between BigFLICA (nIC=750) and 3,913 IDPs in the UKB dataset. We excluded an nIDP if both methods have prediction r-value< 0.1.

Variable Names	r-value FLICA	-log10(p) FLICA	r-value IDPs	-log10(p) IDPs	Percent Improvement	Sample size
**cognitive phenotypes**						
Digits entered correctly (0.1)	0.104	3.6	0.003	0.3	3141.6	1126
Time to complete round (1.2)	0.108	11.7	0.073	5.9	47.4	4119
Number of fluid intelligence questions attempted within time limit (1.0)	0.107	11.6	0.078	6.6	37.6	4116
Duration to first press of snap-button in each round (2.7)	0.11	37.6	0.081	21.1	35.1	13749
Mean time to correctly identify matches (0.0)	0.108	38.6	0.083	23.1	30.6	14471
Duration screen displayed (2.0)	0.103	33.6	0.079	20.3	30.2	13809
Duration to first press of snap-button in each round (2.10)	0.104	33.7	0.08	20.3	30.1	13751
Duration to complete alphanumeric path (trail #2) (0.0)	0.145	33.7	0.113	20.8	28.7	6966
Time to complete round (0.2)	0.113	41	0.088	25.5	27.8	14115
Number of symbol digit matches made correctly (0.0)	0.156	43.5	0.122	27	27.7	7862
Number of symbol digit matches attempted (0.0)	0.167	49.8	0.132	31.4	26.6	7862
Number of fluid intelligence questions attempted within time limit (2.0)	0.151	68.4	0.122	44.9	23.8	13362
Duration to first press of snap-button in each round (2.11)	0.1	31.6	0.082	21.3	22.9	13741
Time to complete round (0.1)	0.143	36	0.117	24.4	22.5	7670
Time to complete round (2.2)	0.114	39.4	0.093	26.8	22.1	13394
Mean time to correctly identify matches (2.0)	0.141	61.6	0.116	42	21.6	13768
Maximum digits remembered correctly (0.0)	0.15	38.2	0.129	28.7	15.9	7465
Fluid intelligence score (2.0)	0.256	198.7	0.232	162.6	10.3	13362
Fluid intelligence score (0.0)	0.199	47.8	0.183	40.5	8.8	5266
Touchscreen duration (2.0)	0.127	52.5	0.121	48	4.7	14412
Fluid intelligence score (1.0)	0.18	31	0.188	33.5	-3.9	4116
Number of fluid intelligence questions attempted within time limit (0.0)	0.098	12.4	0.103	13.4	-4	5266
Fluid intelligence score (0.0)	0.198	72.1	0.209	80.5	-5.3	8090
Touchscreen duration (1.0)	0.103	10.8	0.127	16	-19.2	4134
**Health and Medical History Health Outcomes**						
Age asthma diagnosed (0.0)	0.122	5.6	0.051	1.5	139.4	1397
Interpolated Age of participant when non-cancer illness first diagnosed (0.3)	0.102	5.1	0.072	2.9	42.7	1794
Interpolated Year when non-cancer illness first diagnosed (0.3)	0.104	5.3	0.074	3	41.5	1794
Medication for cholesterol, blood pressure, diabetes, or take exogenous hormones (2.0)	0.153	40.4	0.113	22.2	36.4	7507
Treatment/medication code (1140884600 - metformin)	0.121	48.1	0.092	28.1	32	14503
Number of treatments/medications taken (0.0)	0.13	55.6	0.099	32.4	31.8	14503
Age started wearing glasses or contact lenses (2.0)	0.158	73.8	0.124	45.8	27.3	13129
Number of self-reported non-cancer illnesses (0.0)	0.115	43.7	0.093	28.6	24.4	14503
Number of treatments/medications taken (1.0)	0.13	16.7	0.107	11.7	21.4	4134
Age started wearing glasses or contact lenses (0.0)	0.149	61.6	0.124	43.2	19.8	12321
Number of treatments/medications taken (2.0)	0.188	114.4	0.157	80.2	19.4	14435
Treatment/medication code (1140879802 - amlodipine)	0.113	41.9	0.096	30.5	17.8	14503
Medication for cholesterol, blood pressure or diabetes (0.0)	0.15	35.1	0.131	26.7	15.1	6756
Diabetes diagnosed by doctor (2.0)	0.124	49.6	0.108	38.3	14.2	14379
Non-cancer illness code, self-reported (1220 - diabetes)	0.109	39.1	0.098	31.8	11.1	14503
Diagnoses - secondary ICD10 (I10 - I10 Essential (primary) hypertension)	0.146	69.8	0.135	59.5	8.4	14503
Overall health rating (0.0)	0.113	41.6	0.104	35.7	8.1	14477
Non-cancer illness code, self-reported (1065 - hypertension)	0.218	155.7	0.203	133.6	7.9	14503
Medication for cholesterol, blood pressure or diabetes (2.0)	0.145	33.2	0.136	29.3	6.7	6829
Overall health rating (2.0)	0.11	39.5	0.113	41.7	-2.8	14388
Non-cancer illness code, self-reported (1261 - multiple sclerosis)	0.111	40.2	0.118	46	-6.6	14503

Table A.3: Comparison of prediction performance of 158 nIDPs between FLICA (nIC=100) and 5,812 IDPs in the HCP dataset. We excluded an nIDP if both methods have prediction r-value< 0.1. The meanings of each variables can be found at HCP wiki: `https://wiki.humanconnectome.org/display/PublicData/HCP+Data+Dictionary+Public-+Updated+for+the+1200+Subject+Release`

Variable Names	r FLICA	-log10(p)	r IDPs	-log10(p)	Improvement (%)	nSubj	Variable Names	r FLICA	-log10(p)	r IDPs	-log10(p)	Improvement (%)	nSubj
Odor_AgeAdj	0.108	3.5	0.036	0.9	197.4	1001	ASR_Thot_Pct	0.216	11.6	0.206	10.6	4.9	1000
FamHist_Fath_DrgAlc	0.158	6.6	0.065	1.7	144.1	999	PicSeq_AgeAdj	0.301	22	0.29	20.4	3.9	1003
NEOFAC_C	0.176	8	0.084	2.4	110	1001	Avg_Weekend_Beer_Wine_Cooler_7days	0.307	22.5	0.296	20.9	3.8	985
ASR_Soma_Pct	0.124	4.4	0.074	2	66.3	1000	DSM_Somp_Pct	0.12	4.1	0.115	3.9	3.6	1000
ASR_Soma_Raw	0.162	6.9	0.101	3.2	60.7	1000	PicSeq_Unadj	0.293	20.9	0.284	19.6	3.2	1003
VSPLOT_CRTE	0.13	4.7	0.085	2.4	53.2	1000	ASR_Oth_Raw	0.168	7.3	0.163	7	2.7	1000
ASR_Intn_Raw	0.101	3.2	0.067	1.8	51.4	1000	ASR_Thot_Raw	0.231	13.2	0.226	12.6	2.4	1000
DSM_Inat_Raw	0.161	6.8	0.109	3.5	48.3	1000	VSPLOT_OFF	0.417	42.8	0.407	40.8	2.3	1000
SSAGA_Alc_D4_Dp_Sx	0.204	10.4	0.139	5.3	46.6	1002	SSAGA_Alc_Hvy_Frq_Drk	0.244	13.9	0.239	13.4	2	951
AngHostil_Unadj	0.103	3.2	0.072	2	42	1002	Avg_Weekend_Cigarettes_7days	0.181	8.3	0.178	8	1.9	985
ProcSpeed_AgeAdj	0.24	14.2	0.175	7.9	37.2	1003	SSAGA_Alc_Hvy_Max_Drinks	0.466	52	0.459	50.3	1.5	951
DSM_Depr_Raw	0.118	4.1	0.087	2.5	36.7	1000	ASR_Extn_T	0.186	8.8	0.183	8.6	1.4	1000
Times_Used_Any_Tobacco_Today	0.157	6.5	0.117	3.9	34.8	985	NEOFAC_N	0.159	6.6	0.157	6.5	0.9	1001
ProcSpeed_Unadj	0.268	17.6	0.201	10.2	33.5	1003	ASR_Rule_Raw	0.285	19.7	0.283	19.4	0.6	1000
ASR_Intr_Raw	0.189	9.1	0.143	5.5	32.3	1000	ER40SAD	0.117	4	0.117	4	0.6	1000
SSAGA_Mj_Ab_Dep	0.127	4.5	0.097	3	31.2	1002	SSAGA_Alc_Hvy_Frq_5plus	0.3	20.8	0.299	20.6	0.5	951
SSAGA_Alc_D4_Ab_Sx	0.153	6.3	0.119	4.1	29.4	1002	ListSort_Unadj	0.397	38.7	0.4	39.3	-0.7	1003
NEOFAC_O	0.253	15.6	0.195	9.6	29.2	1001	AngAggr_Unadj	0.28	19	0.283	19.4	-1.1	1002
Flanker_AgeAdj	0.181	8.4	0.142	5.5	27.5	1003	VSPLOT_TC	0.361	31.8	0.367	32.9	-1.7	1000
ASR_Intr_Pct	0.161	6.8	0.127	4.5	26.8	1000	FearAffect_Unadj	0.118	4.1	0.12	4.2	-1.9	1002
DDisc_AUC_40K	0.341	28.3	0.277	18.7	23	1000	Total_Drinks_7days	0.28	18.7	0.286	19.5	-2.1	985
SSAGA_Alc_12_Drinks_Per_Day	0.336	26	0.277	17.7	21.3	950	NEOFAC_A	0.223	12.4	0.229	12.9	-2.3	1001
Flanker_Unadj	0.239	14	0.197	9.8	20.9	1003	Total_Cigarettes_7days	0.174	7.7	0.178	8	-2.4	985
SSAGA_Times_Used_Stimulants	0.117	4	0.098	3	19.9	1002	ListSort_AgeAdj	0.393	38.1	0.406	40.6	-3	1003
ASR_Witd_Raw	0.23	13	0.192	9.3	19.5	1000	DSM_Hype_Raw	0.163	6.9	0.17	7.5	-4	1000
PicVocab_Unadj	0.572	87.7	0.487	60.5	17.4	1003	Avg_Weekday_Any_Tobacco_7days	0.183	8.5	0.191	9.1	-4.3	985
ER40ANG	0.102	3.2	0.087	2.5	16.9	1000	Avg_Weekend_Drinks_7days	0.265	16.8	0.278	18.5	-4.8	985
Avg_Weekday_Drinks_7days	0.197	9.7	0.169	7.3	16.8	985	SSAGA_Alc_12_Frq	0.228	12.3	0.24	13.5	-4.8	951
EmotSupp_Unadj	0.128	4.6	0.11	3.6	16.5	1002	ASR_Rule_Pct	0.185	8.7	0.194	9.5	-5	1000
Dexterity_AgeAdj	0.316	24.3	0.271	18	16.5	1003	ASR_Aggr_Raw	0.103	3.2	0.109	3.5	-5.3	1000
SSAGA_Alc_Hvy_Drinks_Per_Day	0.335	25.9	0.288	19.1	16.3	950	THC	0.264	17	0.281	19.2	-6	1003
CardSort_AgeAdj	0.345	28.9	0.297	21.4	16	1001	Total_Any_Tobacco_7days	0.192	9.2	0.205	10.4	-6.2	985
PicVocab_AgeAdj	0.57	87	0.492	61.9	15.8	1003	Avg_Weekday_Cigarettes_7days	0.167	7.2	0.178	8.1	-6.3	985
Taste_AgeAdj	0.186	8.8	0.161	6.8	15.6	998	SSAGA_Times_Used_Illicits	0.147	5.8	0.158	6.6	-6.5	1002
DSM_Antis_Pct	0.168	7.3	0.146	5.7	15.4	1000	PercStress_Unadj	0.165	7.1	0.177	8.1	-6.7	1002
SSAGA_Times_Used_Hallucinogens	0.163	7	0.143	5.6	14.1	1002	SSAGA_Alc_12_Frq_5plus	0.324	24.3	0.349	28.1	-7.1	951
DSM_Adh_Pct	0.219	11.9	0.192	9.3	14.1	1000	ASR_Crit_Raw	0.185	8.7	0.2	10.1	-7.5	1000
Taste_Unadj	0.188	9	0.165	7.1	13.9	998	MMSE_Score	0.175	7.9	0.189	9.1	-7.6	1003
ASR_Attn_Raw	0.147	5.8	0.129	4.7	13.8	1000	Num_Days_Used_Any_Tobacco_7days	0.218	11.8	0.238	14	-8.7	1000
Dexterity_Unadj	0.302	22.2	0.266	17.3	13.5	1003	PercHostil_Unadj	0.107	3.4	0.117	4	-8.8	1002
SSAGA_Alc_12_Max_Drinks	0.409	39.2	0.36	30.1	13.5	951	SCPT_SPEC	0.185	8.7	0.204	10.5	-9.7	1000
DDisc_AUC_200	0.295	21.2	0.26	16.5	13.4	1000	ER40_CR	0.158	6.6	0.176	8	-10.4	1000
DSM_Depr_Pct	0.119	4.1	0.105	3.4	13.4	1000	ASR_Totp_Raw	0.151	6.1	0.171	7.6	-11.8	1000
SSAGA_ChildhoodConduct	0.192	9.4	0.169	7.5	13.4	1002	SSAGA_TB_Smoking_History	0.148	5.9	0.168	7.4	-11.8	1002
ASR_Witd_Pct	0.151	6.1	0.134	4.9	13.1	1000	IWRD_TOT	0.174	7.8	0.198	9.9	-12.1	1000
CardSort_Unadj	0.36	31.6	0.319	24.7	12.9	1001	ASR_TAO_Sum	0.169	7.5	0.195	9.6	-13.1	1000
SSAGA_Alc_Hvy_Frq	0.254	15.1	0.226	12.1	12.5	951	Num_Days_Drank_7days	0.183	8.6	0.211	11.1	-13.3	1000
PMAT24_A_CR	0.512	67.4	0.456	51.9	12.3	999	Avg_Weekend_Any_Tobacco_7days	0.193	9.3	0.224	12.2	-13.6	985
LifeSatisf_Unadj	0.208	10.8	0.186	8.8	11.8	1002	FearSomat_Unadj	0.106	3.4	0.125	4.4	-15.1	1002
DSM_Somp_Raw	0.154	6.3	0.138	5.2	11.3	1000	ASR_Totp_T	0.153	6.2	0.183	8.5	-16.1	1000
DSM_Adh_Raw	0.182	8.5	0.165	7.1	10.4	1000	DSM_Anxi_Raw	0.143	5.6	0.174	7.8	-17.6	1000
Avg_Weekday_Beer_Wine_Cooler_7days	0.286	19.6	0.26	16.3	10	985	SSAGA_TB_Still_Smoking	0.181	8.4	0.225	12.5	-19.5	1002
PSQI_Score	0.168	7.4	0.153	6.3	9.9	1003	SCPT_SEN	0.083	2.4	0.106	3.4	-21.2	1000
Total_Beer_Wine_Cooler_7days	0.36	31.1	0.329	25.9	9.5	985	SSAGA_Mj_Times_Used	0.172	7.7	0.221	12.1	-21.9	1002
ASR_Attn_Pct	0.188	9	0.173	7.7	8.8	1000	MeanPurp_Unadj	0.085	2.4	0.11	3.6	-22.8	1002
SSAGA_Alc_12_Frq_Drk	0.275	17.5	0.257	15.3	7.1	951	PercReject_Unadj	0.083	2.4	0.108	3.5	-23.4	1002
ReadEng_AgeAdj	0.494	62.3	0.464	54.4	6.3	1003	NEOFAC_E	0.102	3.2	0.139	5.3	-26.8	1001
DSM_Antis_Raw	0.244	14.5	0.229	13	6.2	1000	DSM_Anxi_Pct	0.088	2.6	0.122	4.3	-27.7	1000
ASR_Extn_Raw	0.203	10.3	0.191	9.3	6	1000	Avg_Weekend_Hard_Liquor_7days	0.074	2	0.106	3.4	-29.8	985
ASR_Aggr_Pct	0.119	4.1	0.113	3.7	5.6	1000	SSAGA_Alc_Age_1st_Use	0.136	4.9	0.202	9.8	-32.8	951
ReadEng_Unadj	0.487	60.4	0.464	54.2	5	1003	Total_Hard_Liquor_7days	0.07	1.9	0.135	4.9	-47.9	985

Table A.4: Three examples of top 10 most significant correlations of BigFLICA modes (left) and IDPs (right) with nIDPs in UKB dataset.

BigFLICA modes	r-value	p-value	IDP names	r-value	p-value
**Top 10 modes/IDPs correlate with *fluid intelligence***					
IC25	-0.146	6.54E-65	IDP_tfMRI_90th-percentile_BOLD_shapes	-0.074	1.05E-15
IC57	-0.122	3.49E-45	IDP_tfMRI_median_BOLD_shapes	-0.069	1.28E-13
IC332	-0.081	7.26E-21	IDP_tfMRI_90th-percentile_zstat_faces-shapes_amygdala	0.068	1.48E-13
IC484	-0.072	9.87E-17	rfMRI amplitudes (ICA25 node 6)	0.066	2.75E-13
IC4	-0.069	1.18E-15	rfMRI connectivity (ICA100: IC13-IC32)	-0.065	3.82E-13
IC27	0.064	1.98E-13	IDP_tfMRI_90th-percentile_zstat_shapes	-0.063	7.95E-12
IC188	-0.058	2.80E-11	IDP_tfMRI_median_zstat_faces-shapes	0.063	1.20E-11
IC708	-0.055	1.51E-10	IDP_tfMRI_median_zstat_faces-shapes_amygdala	0.062	1.51E-11
IC164	0.055	1.68E-10	rfMRI connectivity (ICA100: IC11-IC19)	0.056	3.94E-10
IC47	-0.054	3.40E-10	IDP_tfMRI_median_BOLD_faces-shapes	0.057	5.16E-10
**Top 10 modes/IDPs correlate with *Age started wearing glasses or contact lenses***					
IC164	0.101	2.74E-31	IDP_tfMRI_90th-percentile_BOLD_faces-shapes	0.081	5.38E-18
IC25	0.067	1.63E-14	IDP_tfMRI_median_BOLD_faces-shapes	0.064	7.16E-12
IC249	0.057	5.65E-11	IDP_tfMRI_median_zstat_faces-shapes	0.064	1.01E-11
IC13	0.054	7.68E-10	rfMRI connectivity (ICA100: IC4-IC40)	-0.061	1.81E-11
IC138	0.041	2.33E-06	rfMRI connectivity (ICA100: IC17-IC42)	-0.060	4.34E-11
IC190	0.038	1.65E-05	IDP_tfMRI_90th-percentile_zstat_faces-shapes	0.061	9.88E-11
IC563	-0.037	1.84E-05	rfMRI amplitudes (ICA25 node 19)	0.056	8.97E-10
IC656	0.037	1.98E-05	rfMRI amplitudes (ICA100 node 16)	0.054	3.08E-09
IC297	-0.037	2.32E-05	rfMRI connectivity (ICA100: IC15-IC43)	-0.050	3.14E-08
IC580	0.037	2.53E-05	rfMRI connectivity (ICA100: IC21-IC28)	0.049	6.90E-08
**Top 10 modes/IDPs correlate with *hypertension***					
IC259	0.121	2.95E-48	IDP_dMRI_TBSS_MD_External_capsule_L	0.135	4.17E-53
IC38	-0.111	3.09E-41	IDP_dMRI_TBSS_MD_External_capsule_R	0.132	6.44E-51
IC319	0.094	5.98E-30	IDP_dMRI_TBSS_L1_External_capsule_L	0.130	2.29E-49
IC29	0.091	5.82E-28	IDP_dMRI_TBSS_L3_External_capsule_R	0.126	6.51E-47
IC40	0.088	2.82E-26	IDP_dMRI_TBSS_L3_External_capsule_L	0.126	1.70E-46
IC1	-0.083	1.57E-23	IDP_dMRI_TBSS_L3_Anterior_limb_of_internal_capsule_L	0.122	4.64E-44
IC171	-0.073	1.00E-18	IDP_dMRI_TBSS_L1_External_capsule_R	0.122	5.37E-44
IC26	-0.067	6.73E-16	IDP_dMRI_TBSS_ISOVF_External_capsule_L	0.122	7.49E-44
IC176	-0.062	6.75E-14	IDP_dMRI_TBSS_L2_Anterior_limb_of_internal_capsule_L	0.120	2.60E-42
IC84	0.057	9.14E-12	IDP_dMRI_TBSS_MD_Anterior_limb_of_internal_capsule_L	0.118	2.91E-41
**Top 10 modes/IDPs correlate with *handedness***					
IC235	-0.226	5.71E-168	rfMRI connectivity (ICA100: IC29-IC34)	0.115	1.30E-40
IC408	-0.079	1.76E-21	rfMRI connectivity (ICA25: IC1-IC6)	0.095	6.93E-28
IC569	0.066	1.90E-15	rfMRI connectivity (ICA100: IC10-IC34)	0.095	7.70E-28
IC382	0.051	8.40E-10	rfMRI connectivity (ICA25: IC14-IC22)	0.085	7.43E-23
IC251	0.047	1.13E-08	rfMRI connectivity (ICA100: IC3-IC19)	0.085	8.01E-23
IC232	0.043	2.12E-07	rfMRI connectivity (ICA100: IC14-IC34)	0.081	7.28E-21
IC742	0.042	4.41E-07	rfMRI connectivity (ICA25: IC1-IC22)	0.080	2.93E-20
IC643	-0.039	2.39E-06	rfMRI connectivity (ICA100: IC30-IC34)	-0.074	1.46E-17
IC419	0.036	1.66E-05	rfMRI connectivity (ICA100: IC27-IC52)	0.073	3.07E-17
IC332	0.036	1.76E-05	rfMRI connectivity (ICA100: IC6-IC13)	0.071	3.39E-16

Table A.5: Percent of shared variance (%) of BigFLICA decomposition across a range of dimensionalities in the UKB data. Upper triangle: the explained variance of a lower-dimensional decomposition by a higher-dimensional decomposition. Lower triangle: the explained variance of a higher-dimensional decomposition by a lower-dimensional decomposition.

	**IC25**	**IC100**	**IC250**	**IC500**	**IC750**
**IC25**	100.00	99.98	99.99	99.99	99.99
**IC100**	88.57	100.00	99.97	99.98	99.99
**IC250**	86.38	96.99	100.00	99.98	99.99
**IC500**	87.77	95.45	96.79	100.00	99.97
**IC750**	85.33	95.31	96.91	99.65	100.00

Table A.6: A description of 47 Modalities of UKB dataset used in this paper.

Abbreviation	full description
rest k (k=1-25)	Dual regression between IC k of 25 dimensional decomposition of rsfMRI and the whole brain
task z1	Z-statistics of emotion task contrast "shapes"
task z2	Z-statistics of emotion task contrast "face"
task z5	Z-statistics of emotion task contrast "faces>shapes"
task c1	Contrasts of parameter estimate of emotion task contrast "shapes"
task c2	Contrasts of parameter estimate of emotion task contrast "face"
task c5	Contrasts of parameter estimate of emotion task contrast "faces>shapes"
TBSS-FA	Tract-Based Spatial Statistics - fractional anisotropy
TBSS-MD	Tract-Based Spatial Statistics - mean diffusivity
TBSS-MO	Tract-Based Spatial Statistics - tensor mode
TBSS-L1	Tract-Based Spatial Statistics - amount of diffusion along the principal directions 1
TBSS-L2	Tract-Based Spatial Statistics - amount of diffusion along the principal directions 2
TBSS-L3	Tract-Based Spatial Statistics - amount of diffusion along the principal directions 3
TBSS-OD	Tract-Based Spatial Statistics - orientation dispersion index
TBSS-ICVF	Tract-Based Spatial Statistics - intra-cellular volume fraction
TBSS-ISOVF	Tract-Based Spatial Statistics - isotropic or free water volume fraction
tracts	summed tractography map of 27 tracts from AutoPtx in FSL
VBM	voxel-based morphometry
Area	Cortical surface area from Freesurfer
Thickness	Cortical surface thickness from Freesurfer
Jacobian	Jacobian map of nonlinear registration of T1 image to MNI152 standard space
swMRI	T2* image derived from swMRI
T2 lesion	White matter hyperintensity map estimated by BIANCA
